# Efficient *ex vivo* expansion of conserved element vaccine-specific CD8+ T-cells from SHIV-infected, ART-suppressed nonhuman primates

**DOI:** 10.3389/fimmu.2023.1188018

**Published:** 2023-05-03

**Authors:** Sandra Dross, Rasika Venkataraman, Shabnum Patel, Meei-Li Huang, Catherine M. Bollard, Margherita Rosati, George N. Pavlakis, Barbara K. Felber, Katharine J. Bar, George M. Shaw, Keith R. Jerome, James I. Mullins, Hans-Peter Kiem, Deborah Heydenburg Fuller, Christopher W. Peterson

**Affiliations:** ^1^Department of Microbiology, University of Washington, Seattle, WA, United States; ^2^Washington National Primate Research Center, Seattle, WA, United States; ^3^Division of Clinical Research, Fred Hutchinson Cancer Center, Seattle, WA, United States; ^4^Center for Cancer and Immunology Research, Children’s National Hospital and Department of Pediatrics, The George Washington University, Washington, DC, United States; ^5^Department of Laboratory Medicine and Pathology, University of Washington, Seattle, WA, United States; ^6^Human Retrovirus Section, Vaccine Branch, National Cancer Institute at Frederick, Frederick, MD, United States; ^7^Human Retrovirus Pathogenesis Section, Vaccine Branch, National Cancer Institute at Frederick, Frederick, MD, United States; ^8^Perelman School of Medicine, University of Pennsylvania, Philadelphia, PA, United States; ^9^Division of Vaccine and Infectious Diseases, Fred Hutchinson Cancer Center, Seattle, WA, United States; ^10^Department of Medicine, University of Washington, Seattle, WA, United States; ^11^Department of Global Health, University of Washington, Seattle, WA, United States

**Keywords:** HIV-1 cure, therapeutic vaccines, DNA vaccine, antigen-specific T cells, rhesus macaque, nonhuman primate models, cell therapies

## Abstract

HIV-specific T cells are necessary for control of HIV-1 replication but are largely insufficient for viral clearance. This is due in part to these cells’ recognition of immunodominant but variable regions of the virus, which facilitates viral escape *via* mutations that do not incur viral fitness costs. HIV-specific T cells targeting conserved viral elements are associated with viral control but are relatively infrequent in people living with HIV (PLWH). The goal of this study was to increase the number of these cells *via* an *ex vivo* cell manufacturing approach derived from our clinically-validated HIV-specific expanded T-cell (HXTC) process. Using a nonhuman primate (NHP) model of HIV infection, we sought to determine i) the feasibility of manufacturing *ex vivo*-expanded virus-specific T cells targeting viral conserved elements (CE, CE-XTCs), ii) the *in vivo* safety of these products, and iii) the impact of simian/human immunodeficiency virus (SHIV) challenge on their expansion, activity, and function. NHP CE-XTCs expanded up to 10-fold following co-culture with the combination of primary dendritic cells (DCs), PHA blasts pulsed with CE peptides, irradiated GM-K562 feeder cells, and autologous T cells from CE-vaccinated NHP. The resulting CE-XTC products contained high frequencies of CE-specific, polyfunctional T cells. However, consistent with prior studies with human HXTC and these cells’ predominant CD8^+^ effector phenotype, we did not observe significant differences in CE-XTC persistence or SHIV acquisition in two CE-XTC-infused NHP compared to two control NHP. These data support the safety and feasibility of our approach and underscore the need for continued development of CE-XTC and similar cell-based strategies to redirect and increase the potency of cellular virus-specific adaptive immune responses.

## 1 Introduction

While HIV-specific T cells are necessary for viral control, they are unable to suppress virus to undetectable levels in most individuals and are insufficient to clear infection (1, 2). HIV-specific T cells often target the variable, non-conserved regions of the virus, allowing viral mutation and immune escape without substantial fitness cost (3). We (4–9) and other investigators (10) have employed different vaccine strategies to redirect the T-cell response towards conserved viral regions. Our Conserved Elements (CE) approach is distinct in that it employs immunogens determined by both sequence conservation analyses and associations with viral control. Specifically, we previously developed CE HIV DNA vaccines comprised of 7 conserved regions within p24Gag and both these p24Gag CE plus 12 conserved regions in Env (4, 5, 8, 11, 12) and evaluated their immunogenicity and efficacy in nonhuman primate (NHP) models of AIDS (4, 6, 13). In rhesus macaques immunized with a DNA vaccine expressing an SIV analog of HIV CE and/or acutely infected with SIV, CE-specific T-cell responses comprised a substantial 1% of the total circulating CD3^+^ cell population after only 2-3 immunizations (13, 14). Furthermore, following SIV challenge, we found that higher levels of CE-specific T-cell responses were associated with improved viral control during acute infection (13, 14).

The goal in this study was to increase the frequency of CE-specific T-cell responses *in vivo* by first expanding CE-specific T cells *ex vivo*. We have shown that *ex vivo* antigen presentation by peptide-pulsed dendritic cells and PHA-stimulated peripheral blood mononuclear cells (PBMCs) efficiently expands antigen-specific T cells, generating both *de novo* T-cell responses and amplifying existing antigen-specific T-cell subsets (15). For individuals with CMV infection, this process was previously shown to efficiently expand virus-specific T cells and was associated with prolonged periods of disease-free survival and complete remission (15–17). An analogous therapy prevented relapse after hematopoietic stem cell transplantation in patients with B cell- or T cell-derived, EBV-associated lymphoma or lymphoproliferative disorders (18). As a cell-based therapy for HIV-1 infection, we have shown that HIV-specific T cells can be efficiently expanded from, and safely infused into ART-suppressed participants living with HIV-1 (19). Similar products can be generated from uninfected, antigen-naïve donors (20, 21). Here, we applied this knowledge to build an NHP model of adoptively transferred antigen-specific T-cell therapy. We hypothesized that enriched CE-expanded T cells (CE-XTC) could be manufactured with similar efficiency and augment adaptive immune responses *in vivo* following challenge with simian/human immunodeficiency virus (SHIV). We redirected HIV-specific immune responses in both uninfected and SHIV-infected macaques to target conserved epitopes *via* CE vaccination, expanded these potent antigen-specific T cells *ex vivo*, and then reinfused each product into the autologous host. We achieved the goal of increasing the frequency of T cells targeting conserved viral sequences, observing large (median: 61-fold) increases in the frequency of CE-specific T cells during *ex vivo* expansion. Our novel NHP model furthermore establishes the safety of CE-XTC and recapitulates limitations that have been observed in early-stage clinical trials, providing proof-of-principle for further optimization in the preclinical setting to achieve greater persistence. These findings open new doors to merge cell therapy with other gene therapy strategies to protect cells against infection and/or enhance their virus-specific function, e.g., through the use of CRISPR-Cas9 gene editing and/or virus-specific chimeric antigen receptors.

## 2 Methods

### 2.1 Study design and ethics statement

Juvenile male Indian-origin rhesus macaques (Macaca mulatta, n=9) were used in this study. Five animals were infected with the clade C simian/human immunodeficiency virus SHIV-1157ipd3N4 (“SHIV”) (22) 34 weeks prior to vaccination and placed on ART (tenofovir disoproxil fumarate, 5.1 mg/mL; emtricitabine, 40mg/mL; and dolutegravir, 2.5mg/mL) 27 weeks prior to vaccination. PBMC from these animals were used to test the impact of suppressed SHIV infection on our CE-XTC manufacturing process. Data from all 5 SHIV-infected, ART-suppressed animals (IDs A17026, A17032, A17035, A17023 and A17025) are included in our manufacturing experiments, whereas cells from only 3 of these 5 animals (IDs A17026, A17032, and A17035) were available for subsequent functional assays. An additional 4 uninfected animals (IDs A17019 and A17020, A17044 and A17045) were used to quantify SHIV acquisition following infusion of CE-XTC: 2 animals were vaccinated then infused with CE-XTC, and compared to 2 non-vaccinated, no-CE-XTC controls. Animal protocols were reviewed and approved by the Institutional Animal Care and Use Committee at the University of Washington (IACUC # 3235-04) and were compliant with the U.S. Department of Health and Human Services *Guide for the Care and Use of Laboratory Animals*.

### 2.2 SHIV conserved elements vaccine design and delivery

**Figure 1:**
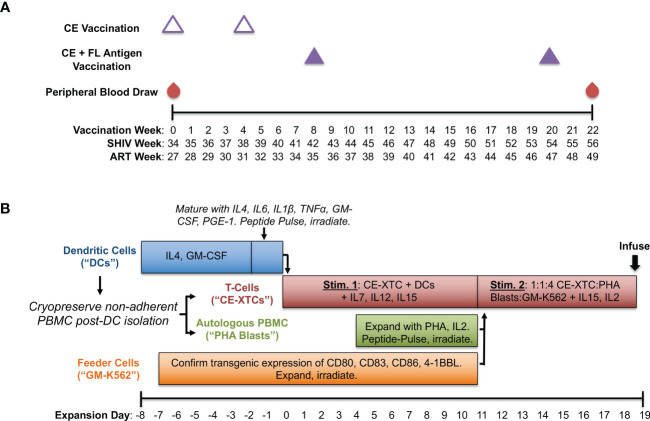
Production of conserved elements (CE) antigen-specific nonhuman primate T-cells (NHP CE-XTCs) from vaccinated macaques. **(A)** Macaques received a CE DNA vaccine on vaccination weeks 0 and 4 (open triangles) followed by two additional doses of the CE DNA vaccine co-delivered with a second plasmid expressing full length (FL) SIV Gag and Env on weeks 8 and 20 (closed purple triangles). NHP CE-XTC expansion began 2 weeks after the final CE vaccine dose with 30-50 mL of peripheral blood. Schedule is also shown relative to weeks post-SHIV infection (“SHIV Week”) and post-antiretroviral therapy (ART) initiation (“ART Week”). **(B)** Following Ficoll separation of week 22 blood draws and a plastic adherence step, the non-adherent fraction was cryopreserved, and an 8-day dendritic cell (DC) culture was initiated (blue bar). NHP CE-XTCs (red bar) were induced by mixing with autologous, CE peptide-pulsed, irradiated DC. DC manufacturing spanned Days -8 through 0, and were added to CE-XTCs on Day 0. Non-adherent PBMC stimulated with phytohemagglutinin (PHA) and IL2 (PHA blasts, green bar) were similarly pulsed with CE peptides and irradiated on Day 11. Finally, GM-K56 cells, typically expanded over a 2-week time course (orange bar), were irradiated and added to the culture along with PHA blasts on Day 11. Day 11 CE-XTC cultures were mixed with irradiated, peptide-pulsed PHA blasts and irradiated GM-K562 feeder cells at a 1:1:4 ratio. This mixed culture was rapidly expanded in G-Rex 100 flasks for at least one week prior to infusion.

To generate SHIV-specific T-cell responses *in vivo*, we used a CE DNA vaccine containing conserved SIV and HIV Gag and Env sequences and full-length (FL) Gag and Env sequences matched to the SHIV used to model ART-suppressed infection in this study (SHIV-1157ipd3N4). As in previous studies, two forms of CE antigens were used, differing by 1 amino acid per p24CE and 1-5 amino acids in Env CE (5, 14). The SHIV.C.CH848 strain used for subsequent intrarectal challenge experiments was nearly identical to the variable Gag and Env sequences in our full-length (FL) vaccine with 100% homology to the conserved element (CE) antigens in Gag and 98.9% in Env. Our vaccine consisted of DNA plasmids encoding highly conserved regions of SIV Gag and HIV Env in addition to FL SIV Gag and HIV Env antigens as previously published (14) with the following modifications: among the full-length HIV Env gp145dID plasmid mixture (composed of HIV BaL gp145dID and HIV 1086 gp145dID) we included clade C SHIV1157 (22), and vaccinations were adjuvanted by a plasmid encoding heat labile enterotoxin LT (23, 24). Briefly, we administered 2 priming doses of CE DNAs alone spaced 4 weeks apart followed by 2 boosting doses of CE + FL DNAs spaced 12 weeks apart (**Figure 1A**). Vaccinations were delivered by particle-mediated epidermal delivery (PMED; also referred to as Gene Gun) as previously described (24) at a dose of 16ug DNA per antigen to the lower abdomen (SIV Gag) and upper thigh proximal to inguinal lymph nodes (HIV Env).

### 2.3 Expansion of conserved elements antigen-specific nonhuman primate T cells

**Figure 2:**
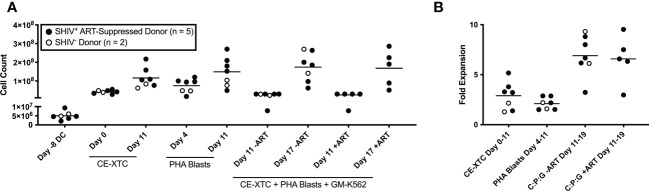
*Ex vivo* expansion of NHP CE-XTCs and supporting cells. NHP CE-XTCs were prepared as described in **Figure 1** and cell counts at each manufacturing phase were determined from SHIV-infected, ART suppressed donors (closed circles, n = 5) and uninfected donors (open circles, n = 2). **(A)** Cell counts from representative manufacturing time points detailed in **Figure 1**, including initiation of DC culture (Day -8 DC), CE-XTCs on Day 0 and Day 11 following addition of CE-peptide pulsed, irradiated DC, PHA blasts on Day 4 (thaw day) and Day 11 (after 7 days of expansion), and co-cultured CE-XTC on Day 11 (addition of expanded PHA blasts and GM-K562) and Day 17 (after 6 days of expansion). “CE-XTC + PHA Blasts + GM-K562” includes comparisons of cell numbers from SHIV-infected, ART-suppressed donors at day 11 and 17 when expanded in the absence (-ART) or presence (+ART) of antiretroviral drugs in the culture media. **(B)** Fold expansion for the CE-XTC, PHA blasts, and CE-XTC/PHA blast/GM-K562 mixed cultures in the absence (-ART) or presence (+ART) during the indicated time interval. C:P:G refers to the final co-culture of CE-XTC, PHA blasts, and GM-K562 feeders. Cells from uninfected donors (animal IDs A17044 and A17045) were only assessed in the absence of ART. In subsequent figures, CE-XTC phenotypic and functional data includes 3 of 5 SHIV-infected, ART-suppressed donors (IDs A17026, A17032, and A17035) but was not available from 2 others (IDs A17023 and A17025).

A schematic of our *ex vivo* CE-XTC manufacturing process is shown in **Figure 1B**. We built on our existing antigen-specific T-cell expansion protocol (25, 26) using peptide pools consisting of SIV Gag and HIV Env CE regions (14), and pulsed dendritic cells (DC) with 2 μg/ml per peptide. These CE peptide pools consisted of both 10 and 12-15 mers overlapping by 1 and 4 amino acids, respectively and were 100% homologous to the CE vaccine sequences. Base DC media (Cell Genix, Portsmouth, NH) contained 2 mM Glutamax (Thermo Fisher, Waltham, MA) and 1% Penicillin/Streptomycin (Thermo Fisher). Base media for CE-XTC and PHA Blasts was 45% Advanced RPMI (Thermo Fisher), 45% Eagle Hank’s Amino Acids Click’s (Irvine Scientific, Santa Ana, CA), and 10% heat-inactivated Human AB serum (Gemini Bio, West Sacramento, CA) plus 2mM GlutaMax. Media cytokines and growth factors were either recombinant rhesus macaque (rrm) generously provided by Dr. Francois Villinger, or recombinant human (rh) from Peptrotech (Rocky Hill, NJ). Antiretroviral medias also contained Indinavir (30 ng/mL) and Raltegravir (15 ng/mL) provided by the NIH AIDS Reagent Program. NHP CE-XTC expansion began with 30-50 mls of peripheral blood drawn 2 weeks post-4th CE vaccination (**Figure 1A**). Following PBMC separation *via* Ficoll and a 2-hour plastic adherence protocol, the non-adherent fraction was cryopreserved in 90% fetal bovine serum (Gemini Bio) + 10% DMSO (Sigma-Aldrich, St. Louis, MO), and the adherent fraction containing DC was cultured in media containing rrmIL-4 (1000 U/mL, and rhGM-CSF (800 U/mL) (**Figure 1B**). Day -8 DC counts (**Figure 2A**) correspond to a bulk cell count of the plastic-adherent fraction. Six days later, DC were matured in media containing rrmIL-4, rhIL-6 (100 ng/mL), rrmIL-1β (10 ng/mL), rrmTNFα (10 ng/mL), rhGM-CSF, and rhPGE-1 (1 ng/mL)(R&D Systems, Minneapolis, MN). Two days later, a portion of the non-adherent cells were thawed. NHP CE-XTCs were induced from this population first by mixing with autologous, matured DCs which had been pulsed with CE peptides and irradiated (25 Gy) immediately prior to co-culture in media containing rhIL-7 (10 ng/mL), rrmIL-12 (10 ng/mL), and rhIL-15 (100 ng/mL). Four days later, the remainder of the non-adherent cells (referred to as PHA blasts) were thawed and stimulated with phytohemagglutinin (PHA)(5 ug/mL, EMD Millipore, Burlington, MA) and rhIL-2 (50 ng/mL). One week later, PHA blasts were pulsed with CE peptides analogously to DC and irradiated (75 Gy). On the same day, GM-K562 cells, which were expanded over a 2-week time course in Advanced RPMI + 10% fetal bovine serum and 1% Penicillin/Streptomycin, were also irradiated (200 Gy). Irradiated GM-K562 cells, a feeder layer designed to further stimulate our CE-XTC cultures, are engineered to express several co-stimulatory markers including CD80, CD83, CD86, and 4-1BBL (19). CE-XTCs (culture day 11) were harvested and mixed with the irradiated, peptide-pulsed PHA blasts, and the irradiated, GM-K562 cells, typically at a 1:1:4 ratio, respectively. Where indicated, antiretrovirals were added at this culture step, consistent with our previous clinical cell manufacturing protocol (19). Finally, this mixture was rapidly expanded in G-Rex 100 flasks (Wilson Wolf) for one week. We evaluated this NHP CE-XTC process in cells from a total of 5 CE-vaccinated animals (3 SHIV-infected and 2 SHIV-uninfected rhesus macaques). NHP CE-XTC products were then administered to the 2 SHIV-uninfected animals *via* the intravenous route, prior to dose-escalating intrarectal SHIV-challenges.

### 2.4 Intracellular staining for T cell CE vaccine responses and surface staining for CFSE+ CE-XTC detection

The frequency of CE-specific T cells (magnitude) and their effector functions (polyfunctionality) were measured by intracellular staining and flow cytometry at 4 sequential time points: pre-vaccination, post-vaccination, post-NHP CE-XTC expansion and at necropsy. Cells were stimulated overnight (10-14 hours) with SIV Gag CE or HIV Env CE peptide pools homologous to the vaccine CE at 1 μg/ml or DMSO negative solvent control. Peptides used in these assays and in our CE-XTC manufacturing process were homologous to our CE vaccine. 1 μg/mL brefeldin A was added 1 hour into stimulation. Cells were surface stained with CD45 PECF594 (d058-1283, BD), LIVE/Dead Aqua (ThermoFisher), CD3 BV650 (Sp34-2, BD), CD4 BV605 (OKT4, BioLegend), CD8 (RPA-T8, BioLegend), CD28 BV711 (CD28.2, BioLegend), and CD95 APC-Cy7 (DX2, BioLegend), then permeabilized and fixed using CytoFix/Perm kit (BD Biosciences) then intracellularly stained (ICS) with IL-2 AF700 (MQ1-17H12, BioLegend), TNFα Pe-Cy7 (Mab11, BD), granzyme B BV421 (GB11, BD), and CD107a PeCy5 (eBioH4A3, LifeTech) and either IFNɣ FITC (B27, BD) prior to CE-XTC infusion or IFNɣ BB700 (B27, BD) post CE-XTC infusion, to allow detection of CFSE-labeled CE-XTCs in the DMSO negative control at acquisition. A subset of ICS experiments included 30min of α4β7 PE (A4B7R1, NHPRR), CCR9 APC (112509, R&D) staining at 37c and 2x washes immediately prior to stimulus, overnight incubation and ICS. T-cell functions measured by ICS included expression of IFNγ, IL-2, TNFα, and expression of granzyme B and CD107a post-CE stimulus. Flow cytometric data acquisition was performed on a BD LSR II benchtop cytometer and analyzed using FlowJo^TM^ Version 9.9.5 software (BD).

The frequency of CFSE^+^ CE-XTCs was determined immediately prior and 15 minutes post-infusion at 15min and week 9-12 post-infusion (necropsy). Single cell suspensions from 2 CE-XTC-infused animals were isolated, surface stained for CD45 PECF594 (d058-1283, BD), LIVE/Dead Aqua (ThermoFisher), CD3 BV650 (Sp34-2, BD), CD4 BV605 (OKT4, BioLegend), CD8 (RPA-T8, BioLegend). Flow cytometric data acquisition was performed on a BD LSR II benchtop cytometer and analyzed using FlowJo^TM^ Version 9.9.5 software (BD).

### 2.5 Intrarectal SHIV challenge

To measure the impact of CE-XTC therapy on SHIV acquisition, we compared two uninfected, CE-vaccinated, CE-XTC-infused animals that underwent 2 dose-escalating challenges with SHIV.C.CH848 (27–29), administered 0 and 12 days after cell infusion, to two unvaccinated control animals that did not receive NHP CE-XTCs. Challenges were administered on the same day to all 4 animals. Challenge stocks contained 73 ng/mL SIV p27 Gag by ELISA and 4.42×10^6^ infectious units per mL by TZM-bl assay. Diluted stocks for intrarectal challenge were freshly prepared in RPMI 1640 medium without additives. Each animal received 1 mL doses of 1:80 inoculum on day 0, followed 12 days later by 1 mL doses of 1:40 inoculum. Following SHIV challenge, plasma viral load assays amplified a region of SIV Gag specific to unspliced viral RNA: 5’-GCAGAGGAGGAAATTACCCAGTAC-3’ (forward), 5’-CAATTTTACCCAGGCATTTAATGTT-3’ (reverse), 5’-TGTCCACCTGCCATTAAGCCCGA-3’ (probe).

### 2.6 Statistics

Data were prepared and analyzed using Prism version 8 (GraphPad Software). Analyses, as described in the figure legends, were adjusted for multiple comparison when appropriate; otherwise, an alpha of 0.05 was used to determine significance.

## 3 Results

### 3.1 Conserved elements vaccination and NHP CE-XTC manufacturing

The primary goal of this study was to optimize and benchmark an *ex vivo* manufacturing process for NHP CE-XTC from CE-immunized donors to expand highly polyfunctional CE-specific T cells. We utilized the Indian-origin rhesus macaque model to immunize and induce CE-specific T-cell responses, monitor vaccine-specific responses post-vaccination, and prepare vaccine-specific cell products at precise intervals following vaccination. Five animals (3 SHIV-infected, ART-suppressed and 2 uninfected) were vaccinated prior to NHP CE-XTC expansion with DNA vaccines expressing Gag- and Env-CE at study weeks 0, 4, 8 and 20. A DNA vaccine expressing the full-length (FL) Gag and Env was co-delivered with the Gag and Env-CE DNA vaccines at study weeks 8 and 20 (**Figure 1A**). This regimen was based on a previous study in rhesus macaques that showed priming with CE DNA vaccines and then incorporating FL immunogens into subsequent booster doses increased the frequency of CE specific CD4^+^ and CD8^+^ T-cell responses (14).

We compared our cell manufacturing protocol in both SHIV-infected, ART-suppressed animals as well as uninfected animals to quantify the impact of latent infection and potential immune dysfunction. Expansion of NHP CE-XTCs began two weeks after the last DNA vaccine dose at study week 22 (**Figure 1B**). This process aligned 4 co-culture-based stimulation strategies to specifically expand CE-specific cells: i) primary DC pulsed with CE peptides, ii) PHA blasts pulsed with CE peptides, iii) irradiated GM-K562 feeder cells, and iv) autologous T cells from CE-vaccinated animals. Two successive stimulatory cycles were performed to provide peptide presentation, costimulatory signals, and feeder support, as outlined in **Figure 1B**. The DC, PHA blasts, and autologous T cells were all derived from Ficoll-separated whole blood. Plastic adherence was used to isolate DC, which were initially cultured in the presence of IL-4 and GM-CSF, and then 6 days later IL-6, IL-1B, TNFα, and PGE-1 were added to support and mature DC. Two days after maturation (total of 8 days in culture), the adherent DC were pulsed with CE peptides and irradiated. Separately, aliquots of the non-adherent fraction were collected and used to produce irradiated, peptide-pulsed PHA blasts and GM-K562 feeders. CE peptide-pulsed, irradiated DC provided the 1st CE-XTC stimulus at expansion day 0, while PHA blasts and GM-K562 provided the 2nd stimulus at expansion day 11 (1:1:4 ratio of CE-XTC : PHA blast:GM-K562) (**Figure 1**). This mixed culture was expanded for 9 days prior to infusion into autologous NHP.

### 3.2 NHP CE-XTCs expand ≥10-fold and display high magnitude, polyfunctional CE responses

**Figure 3:**
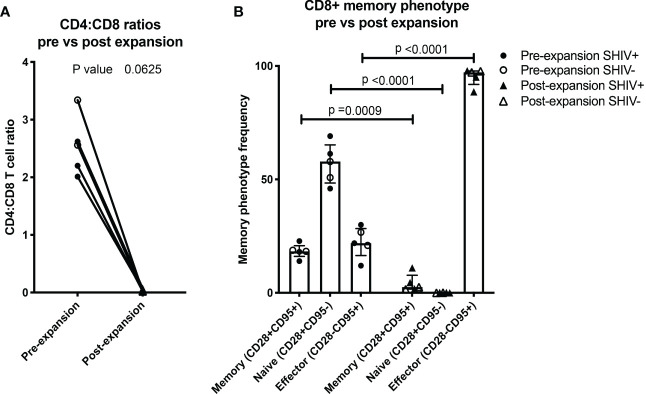
NHP CE-XTC phenotype is predominantly CD8+ effector. **(A)** Pre- (circles) vs. post-CE-XTC expansion CD4:CD8 ratios (triangles) from SHIV-infected, ART suppressed donors (closed shapes, n = 3) and uninfected donors (open shapes, n = 2) demonstrating lack of correlation between CD4:CD8 ratio and donor SHIV status. **(B)** CD8^+^ T cell memory phenotypes, defined by expression of CD8, CD28, and CD95. Statistical comparisons were performed using Wilcoxon matched-pairs signed rank test in panel A and mixed effects model (REML) adjusted for multiple comparisons with Sidak’s multiple comparison tests in panel B.

Throughout the *ex vivo* manufacturing process, we measured total cell counts and the frequency, function, and phenotype of the CE-specific expanded T cells. We focused on 3 time points numbered relative to addition of peptide-pulsed DC (**Figure 1**): Day 0 baseline, day 11 prior to the addition of PHA blasts and GM-K562, and day 17, 6 days after the addition of the PHA blasts and GM-K562 cells. A median 3-fold increase in total viable cell number occurred between day 0 and day 11 of CE-XTC culture and a >5-fold expansion occurred between day 11 and day 17 (**Figure 2**). To address the potential need to suppress SHIV replication in cells from infected, ART-suppressed animals following serial T-cell activation, we compared manufacturing in the presence (+ART) or absence of ART (-ART) in the culture media (19). No differences were observed in NHP CE-XTC yield either due to uninfected/SHIV-infected donor source or in the presence or absence of ART (**Figure 2**). We next quantified CD4^+^ and CD8^+^ T-cell subsets as well as memory and effector phenotypes. Although the percentage of CE-responsive CD4+ and CD8+ T cells increased post-expansion, we observed an increase in CD8^+^ T-cell proportion but a significant concordant decrease in CD4^+^ T-cell proportion within each cell product. Prior to expansion, the median CD4:CD8 ratio was 2.56; whereas post-expansion, the CD4:CD8 ratio was 0.02, i.e., nearly 100% CD8^+^ T cells (**Figure 3A**). Analysis of memory and effector phenotype focused on cell surface expression of CD28 and CD95, demonstrating that CD8^+^ T cells significantly shifted from median frequencies of naïve (57.9%), memory (18.3%), and effector (21.9%) prior to CE-XTC manufacturing to a predominately short-lived CD28-CD95^+^ effector phenotype (median frequencies of naïve 0.07%, memory 2.54%: effector 97.20%) by day 19 of the expansion period (**Figure 3B**). Additionally, CE-XTCs were assessed for mucosal homing markers CCR9 and α4β7, and while CCR9 expression was relatively infrequent (range 1.8-7.2% of all CD8+ CE-XTC), nearly all CE-XTCs expressed α4β7 (range 85.5-98% of all CD8+ CE-XTC). Collectively, these findings show that our manufacturing protocol drove the expansion of CD8^+^ T-cell subsets that were enriched for a differentiated effector phenotype.

**Figure 4:**
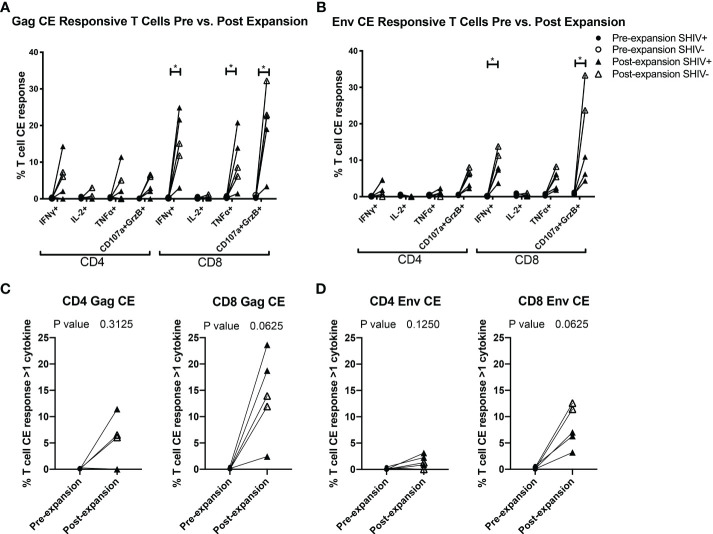
CE-XTC manufacturing induces CE Env- and CE Gag-specific CD4+ and CD8+ T cells expressing one or more effector functions. Pre-expansion PBMC (circles) and post-expansion CE-XTCs (triangles) from SHIV-infected, ART suppressed donors (closed shapes, n = 3) and uninfected donors (open shapes, n = 2) were expanded for 19 days and then stimulated overnight with CE peptide pools comprising either 7 regions of SIV Gag **(A, C)** or 12 regions of HIV Env **(B, D)**. Following peptide stimulation, effector functions were analyzed by intracellular cytokine staining and flow cytometry. Shown are the increases in frequencies of CD4 and CD8 T cells expressing one or more of the cytokines IFN-γ, IL-2, TNFα or co-expression of CD107a and Granzyme B as markers of cytolytic effector function after the 19-day expansion period. P values comparing responses before and after expansion are shown for each individual effector function **(A, B)** or as polyfunctional responses **(C, D)** defined as cells expressing 1 or more cytokine and/or cytolytic effector functions. Individual responses were compared by Mixed-effects model (REML) adjusted for multiple comparisons by Sidak’s multiple comparison test **(A, B)** * = p<0.05. Differences in paired pre- vs. post-expansion T-cell response polyfunctionality were compared by Wilcoxon matched-pairs signed rank test **(C, D)**.

**Figure 5:**
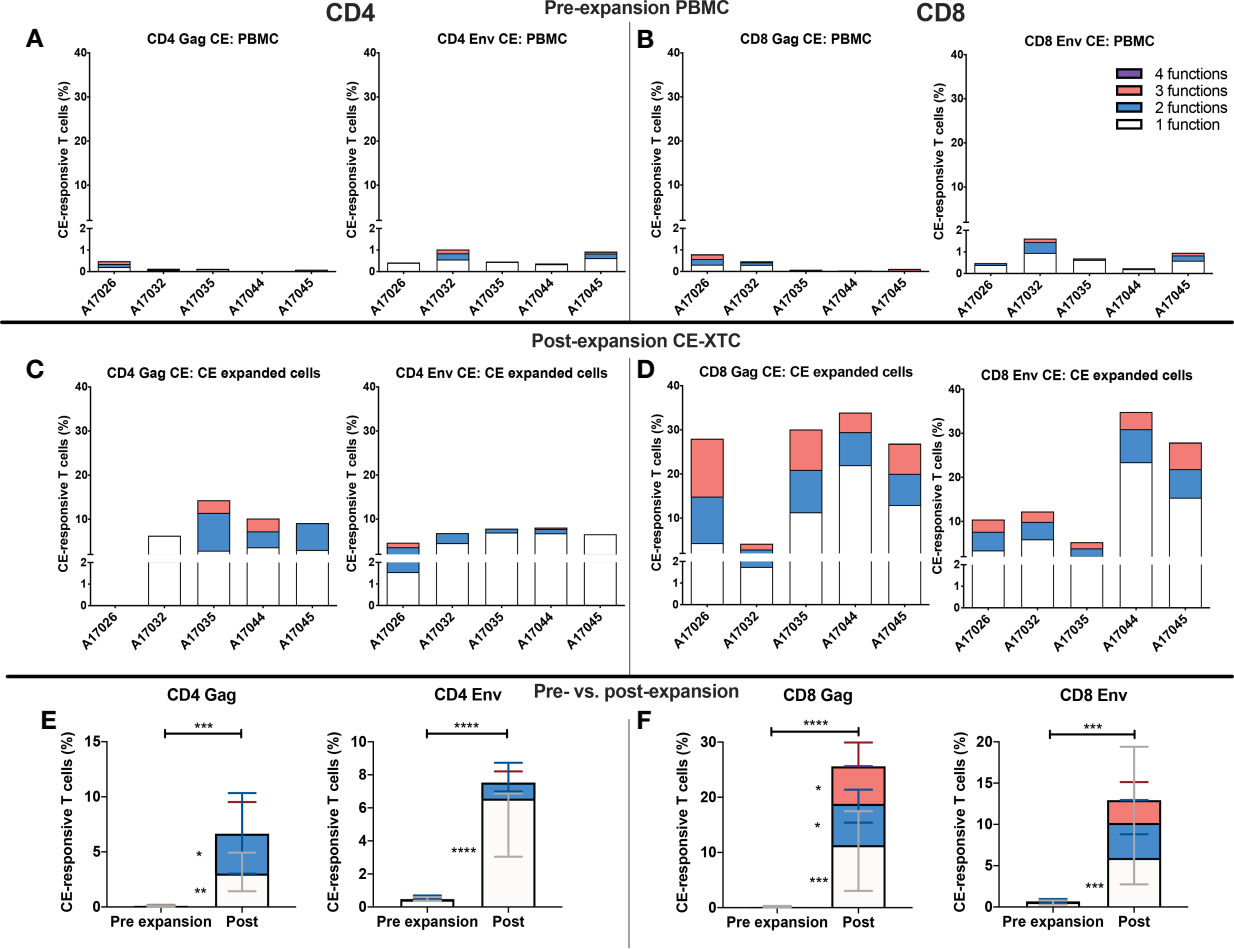
Magnitude and functionality of expanded CE Env- and CE Gag-specific CD4^+^ and CD8^+^ T-cell responses. PBMC or CE-XTCs from all 5 animals were stimulated overnight prior to their expansion and at day 19 post-expansion with overlapping peptide pools representing sequences for each of 7 or 12 conserved elements of SIV Gag and HIV Env, respectively, and their frequencies were measured by flow cytometry following cell surface and intracellular cytokine staining. Shown are the cumulative frequencies of stimulated CD4^+^ **(A, C)** and CD8^+^ **(B, D)** Gag and Env CE-specific T cells expressing IFN-γ, IL-2, TNFα and/or CD107a with granzyme B at pre- **(A, B)** and 19 days post-expansion **(C, D)**. Median 1, 2, 3 and 4 function CE-specific CD4 **(E)** and CD8 **(F)** T cells were compared by mixed-effects model (REML) adjusted for multiple comparisons by Sidak’s multiple comparison test. P <0.05 = *, P <0.01 = **, P < 0.001 = ***, P < 0.0001 = ****.

To further evaluate the function of our CE-XTC cell products, we next measured the frequency of CD4^+^ and CD8^+^ T cells expressing IFN-γ, IL-2, TNFα, and/or co-expression of granzyme B/CD107a. This analysis excludes cytotoxic T cells expressing CD107a that may have degranulated granzyme B prior to peptide stimulation. Prior to vaccination, the frequencies of functional CE-specific T cells in *ex vivo* stimulated cultures were on average below 1% (**Supplementary Figure 1A**). Likewise, *in vivo* analysis of PBMC collected from the CE-vaccinated animals prior to CE-XTC manufacturing showed frequencies of CE-specific CD4^+^ and CD8^+^ T cells in the range of 0.13-1.03% (CD4) and 0.08-1.62% (CD8) that included polyfunctional responses (**Supplementary Figure 1B**). In stark contrast, the magnitude of individual cytokine responses during *ex vivo* CE-XTC expansion increased substantially, with frequencies of IFN-ɣ^+^ and CD107a^+^GranzymeB^+^ cells reaching statistical significance in the CD8^+^ T-cell compartment (**Figures 4A, B**). CD8^+^ CE Env- and CE Gag-specific T cells within each NHP CE-XTC product trended towards an increase in polyfunctionality, whereas CD4^+^ CE-specific T-cell responses exhibited only modest increases in polyfunctionality (**Figures 4C, D**). Single-function, polyfunctional and total magnitude CE Gag and Env responses pre- vs. post-expansion are shown in **Figure 5A, C** for CD4^+^ T-cells and in **Figures 5B, D** for CD8^+^ T-cells, demonstrating that overall magnitude (up to 35% CE^+^ T-cells from animal ID A17044) and individual 1, 2, 3 and 4-function T-cell responses increased post-expansion (**Figures 5E, F**). Significant increases in CE-specific magnitude for both CD4^+^ and CD8^+^ T cells were limited to monofunctional responses to Env peptides, while both monofunctional and polyfunctional responses significantly increased in response to Gag peptides. These results show that our manufacturing process supports the *ex vivo* expansion of CE-XTC up to one order of magnitude, and that expanded cells display increases in polyfunctionality.

### 3.3 Performance of NHP CE-XTC *in vivo*

**Figure 6:**
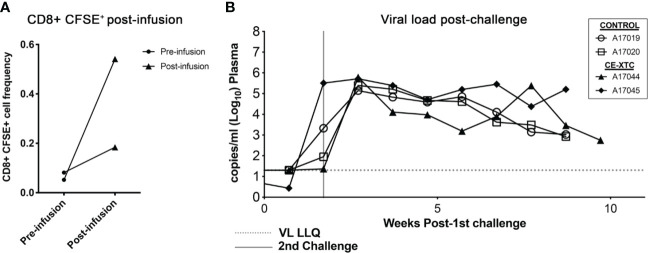
SHIV acquisition following infusion of NHP CE-XTC and subsequent intrarectal challenge with SHIV.C.CH848. **(A)** Manufactured CE-XTCs were labeled with CFSE to facilitate *in vivo* tracking and infused into 2 uninfected juvenile rhesus macaques. Peripheral blood draws were collected immediately prior to CE-XTC infusion and 15 minutes post-infusion and flow cytometry was used to quantify the frequency of CFSE-labeled CD8^+^ T cells. **(B)** Two CE-XTC-infused animals (closed symbols, IDs A17044 and A17045) from panel A along with 2 PBS-infused controls (open symbols, IDs A17019 and A17020) were challenged *via* the intrarectal route on days 0 and 12 post-infusion with low-dose SHIV.CH.848 as described in the *Materials and Methods* section. VL LLQ: Viral load lower limit of quantification. Vertical line represents 2nd challenge dose administered to each animal.

To evaluate the safety and “vein-to-vein” performance of *ex vivo* expanded, vaccine-induced CE-specific T-cell products, we investigated their frequency and biodistribution *in vivo* following autologous infusion, and their ability to protect against intrarectal (IR) SHIV challenge. To specifically track adoptively transferred NHP CE-XTC products and their proliferation *in vivo*, cells were labeled with carboxyfluorescein succinimidyl ester (CFSE) on day 19 of the expansion period, a strategy that is analogous to previous methods (30, 31). The labeled products were then infused into the two CE-vaccinated, uninfected animals, prioritizing a maximum cell dose based on each product’s expansion rather than a standardized cell dose per kilogram body weight. Animal A17044 received a dose of 3.79×10^8^ total cells, while A17045 received 9.6×10^7^ total cells. Using an estimated blood volume of 60 ml/kg in rhesus macaques (32) and CBC data from the day of infusion, we calculated that these infused cell doses translated to 11.27% and 2.8% of total circulating white blood cells for A17044 and A17045, respectively. Peripheral blood samples collected from each animal at 15 minutes post-infusion showed a low CFSE signal over background of 0.2-0.5% of total circulating CD8^+^ T cells (**Figure 6A** and **Supplementary Figure 2**). CE-XTC infusions were safe, with no adverse events observed in either animal post-infusion. Immediately after infusion of NHP CE-XTC, the 2 infused animals and 2 unvaccinated, non-infused controls received a challenge *via* the intrarectal route with SHIV.C.CH848 (27), which has been shown to consistently lead to infection, stable setpoint viremia, and reactivation in ART treatment and interruption studies (28, 29). SHIV.C.CH848 challenge resulted in productive infection in all 4 animals after only 2 challenge exposures spaced 12 days apart. The 2 CE-XTC animals exhibited no measurable differences in SHIV acquisition relative to the 2 controls, and over 9-12 weeks of tracking post-CE-XTC infusion, also showed comparable viral loads (**Figure 6B**). At necropsy, CFSE^+^ CE-XTC cells were not detected in multiple compartments relevant to HIV-1 pathogenesis/persistence, including peripheral blood, mesenteric lymph nodes and upper gastrointestinal tract (**Supplementary Figure 3**). Likewise, no increases in CE-specific T cells or enhanced T cell functionality were observed in CE-XTC infused animals compared to controls in the same compartments (**Supplementary Figure 4**). Consistent with our previous clinical findings investigating this approach for immunotherapy for people living with HIV suppressed on ART (19), these data show that administration of autologous NHP CE-XTC products was safe but was not associated with durable persistence *in vivo*.

## 4 Discussion

Our data demonstrate the feasibility of *ex vivo* NHP CE-XTC manufacturing, including robust expansion of polyfunctional vaccine-induced T cells and safety of these infused products in autologous hosts. CE responses post-vaccination typically comprise <3% of the total CD4^+^ or CD8^+^ population (13, 14). Here, we show that following *ex vivo* expansion in the presence of irradiated GM-K562 feeder cells and autologous, irradiated, peptide-pulsed DC and PHA-blasts, there is a marked increase in the magnitude of CE-specific T cells that are primarily CD8^+^ effector phenotype. We also observed a significant increase in CD8 polyfunctionality and a trending increase in CD4 polyfunctionality. This outcome is consistent with our clinical studies of analogous HIV-specific cell products, referred to as HXTC, which can be efficiently manufactured from seronegative or seropositive human donors (19, 21, 33) using GMP-compliant methodology (20). The NHP CE-XTC process described here was closely modeled after our HXTC process, and the finding that NHP CE-XTC cells are highly polyfunctional and able to secrete higher and multiple cytokine/chemokines is consistent with our previous studies with HXTC cells. Based on our calculations above, the two infusion products were expected to comprise 2.8% and 11.3% of total circulating WBC. Surprisingly, despite the high cell input, within 15 minutes post-infusion, these expanded cells comprised on average of less than 1% of the total CD3^+^ T-cell population and less than 1% of the CD8^+^ T-cell population in the peripheral blood.

The unexpectedly low frequency of infused NHP CE-XTCs is likely one reason for the lack of overt antiviral outcomes, including protection from subsequent mucosal SHIV challenge or enhanced virus control post-acquisition. This result is consistent with our recent clinical trial in which autologous HXTC cells were administered to 6 participants living with HIV (19), where likewise there was no apparent antiviral effect. Interestingly, HXTC cells effectively targeted the viral reservoir in *ex vivo* models (34) but in the clinical trial, infusion of HXTC cells *in vivo* had no impact on viral reservoir size by quantitative viral outgrowth assay and residual plasma viremia (19). While the human study tested HXTC cells as an immunotherapeutic and the NHP study reported here tested this approach as a prophylactic, the parallels in outcomes between this Phase 1 clinical trial and our NHP CE-XTC study reinforce the validity of the NHP model and support its use to iteratively improve autologous therapies in humans. For example, NHPs can be more intensively sampled, which could allow for more detailed kinetic studies to characterize the biodistribution, phenotype, and function of infused cell products. This pilot study is limited by the small number of animals and the use of a rigorous challenge that led to productive infection within only two exposures: this may have overwhelmed potentially efficacious CE-specific T cells. Nevertheless, our results further validate this model to answer key questions and further refine HXTC strategies prior to future studies in NHP and a return to the clinic.

Previous studies of adoptively transferred, antigen-specific lymphocytes in the NHP model contextualize and provide potential paths forward for future CE-XTC experiments. Greene et al. (35) found that lymph node-derived mononuclear cells more efficiently trafficked to secondary lymphoid tissues and liver relative to PBMC (36, 37). Transplantation of PBMC-derived lymphocyte products from donor to MHC-matched recipient animals persisted for at least 2 weeks, suggesting that a graft-versus-host effect could further augment infused cells *in vivo* (36). PBMC-derived SIV-specific CD8^+^ T cells displaying an effector memory phenotype have been shown to persist for up to 24 weeks post-infusion (38). However, these cells targeted immunodominant epitopes that paradoxically may have supported persistence rather than antiviral efficacy, due to virus escape. Intraperitoneal administration of SIV-specific CD8^+^ T cells plus daily IL-2 did not impact the persistence of these cells or lead to antiviral impacts (39). Notably however, proliferation dye-labeled cells persisted in bronchoalveolar lavage samples, suggestive of a non-dividing phenotype which contrasted with expression of canonical activation markers such as CD69 and HLA-DR. Collectively, these studies suggest that our CE-XTC approach could be improved by utilizing tissue-associated lymphocytes for manufacturing (which may present limitations in the clinical setting) and/or applying gene therapy strategies. For example, gene-modified cells could overexpress CXCR5 for homing to B cell follicles (40) or apply lessons from graft-versus-leukemia studies in the cancer field (41) to better understand potential graft-versus-viral reservoir effects (42, 43). We are also interested in cell manufacturing strategies that focus on particularly promising T cell subsets with intrinsic properties associated with *in vivo* persistence, especially T stem cell memory (T_SCM_) which have shown promise both for cancer (44) and PLWH (45).

We expected our 2 NHP infusion products to comprise 2.8% and 11.3% of total circulating WBC, however, this calculation does not account for trafficking to tissues and assumes no cell death. As such, low numbers of circulating cells *in vivo* may also be due to i) insufficient cell dose (9.6×10^7^-3.79×10^8^ total cells), ii) localization of most of the cells to tissues, and/or iii) rapid elimination of the activated effector T cells. Increasing the infused cell dose could be achieved by increasing our initial blood draw volume beyond the 30-50 ml used in this study. Larger blood draw volumes (up to 100 mL based on animal size) and/or multiple sequential blood draws could be applied to administer a larger dose of NHP CE-XTCs. We can also obtain greater numbers and diversity of CE-specific T cells for expansion *via* leukapheresis (46–48). More focused kinetic studies will be required to determine if biodistribution of the infused cells into tissues, including lymph nodes, may have contributed to their low frequency in the blood. Homing of CE-XTC and other adoptively transferred cells to tissues will likely be essential for successful anti-HIV immunotherapies, i.e., to target infected cells more effectively in the tissue-associated viral reservoirs. We have previously shown that homing markers are expressed in clinical HXTC products (19) but do not increase during manufacturing. As such, strategies should be explored to increase the expression of homing markers to enhance trafficking of these cells to tissue sites of HIV acquisition and persistence. Candidate approaches include IL-7 and IL-15 to induce expression of integrin a4b7 expression (49) and CXCR5, respectively (50), and/or transgenic expression of CD62L for lymph node homing (51).

Rapid elimination of the infused cells may be an important factor that contributed to their low frequency in the blood. The majority of the NHP CE-XTCs were CD8^+^ effectors that may have been too short-lived or disproportionately affected by bystander killing that is typical during HIV infection (52). Although these products were not genetically modified to resist infection, it is unlikely that direct infection of the low percentage of CD4^+^ CE-XTC in each infused product contributed to the low frequency of infused cells in the blood or lack of protection against mucosal SHIV challenge. Rather, the predominant short-lived CD8^+^ effectors, combined with insufficient CD4^+^ CE-XTC help, may have led to a T-cell response that was unable to effectively protect against virus challenge. We recently adapted a clinically-derived approach to achieve more balanced CD4:CD8 ratios in adoptively transferred NHP T-cell products (48). Combining this strategy to increase the number of CD4^+^ CE-XTC in our products with culture-based strategies to increase tissue homing markers, e.g., *via* supplementation of culture IL-7 and IL-15 levels, could address each of these limitations. Additionally, as expression of IL-7Rα has been associated with memory potential and CD8^+^ T-cell persistence (53), selective enrichment of IL-7Rα^+^ CD8^+^ T cells may enhance longevity and engraftment of CE-XTC products *in vivo*.

Finally, HXTC and NHP CE-XTC may each be more suited for use in HIV cure approaches that utilize latency reversing agents to augment expression of key reservoir antigens, i.e., as a “shock” to re-activate the latently infected cells combined with an XTC-mediated “kill.” Traditional shock-and-kill hypotheses predict that virus-specific effectors should more effectively control the relatively smaller number of activated infected cells producing virus during latency reversal, compared to a much larger number of infected and virus-producing cells during a *de novo* virus challenge, as used in this study. In this regard, increasing CD4^+^ and CD8^+^ CE-XTC frequencies in both blood and tissues, protecting the CD4^+^ NHP CE-XTC against infection, and/or enhancing their virus-specific function could tip the balance towards stable control of viremia in the absence of suppressive ART. We have investigated numerous approaches to protect CD4^+^ T cells against infection, including expression of peptide-based fusion inhibitors (54) and gene-editing of the HIV coreceptor CCR5 (47, 54, 55). We have also developed strategies to enhance virus-specific immunity *via* short- or long-term expression of chimeric antigen receptors (48, 56, 57) and broadly neutralizing antibodies (58). Finally, we have identified methods to select for virus-specific T cells that could further enrich this key subset, for example, prior to latency reversal therapies or ART interruption (59). In this latter approach, genetic modification strategies could be applied to facilitate small molecule-based selection for a T-cell subset of interest like CE-XTC, effectively increasing their frequency *in vivo*.

In summary, we demonstrate the efficient production of a cell-based therapy to enhance virus-specific T cell immunity that is well tolerated in autologous NHP. As the gold standard preclinical model for HIV prophylaxis and cure, these findings further underscore our NHP model as an important approach to optimize the manufacturing of antigen-specific immune effectors that can prevent virus acquisition and/or control viral rebound following withdrawal of suppressive ART. To enhance the efficacy of this approach, future studies should apply the NHP model in concert with ongoing clinical trials to augment markers associated with cellular persistence, tissue homing, infection resistance, and antiviral potency.

## Data availability statement

The original contributions presented in the study are included in the article/**Supplementary material**. Further inquiries can be directed to the corresponding author.

## Ethics statement

The animal study was reviewed and approved by University of Washington, Protocol #3235-04.

## Author contributions

RV manufactured CE-XTC products with feedback from CP, H-PK, SP and CB. SD performed all flow cytometry and T cell functional assays. M-LH and KJ performed plasma viral load assays. KB and GS produced infectious SHIV.C.CH848 virus and designed the intrarectal challenge experiment. The CE vaccine was designed by JM, MR, GP, and BF. The nonhuman primate vaccination protocol was designed by DF, SD, JM, GP, and BF. H-PK, JM, DF, GP, BF, CP, and SD integrated individual study design components and approved the overall experimental design. SD, CP and DF wrote the manuscript, with critical comments provided by all co-authors. All authors contributed to the article and approved the submitted version.
