# Supplementary material for: Efficient ex vivo expansion of conserved element vaccine-specific CD8+ T-cells from SHIV-infected, ART-suppressed nonhuman primates
Source: Front Immunol. 2023 May 3;14:1188018. doi: 10.3389/fimmu.2023.1188018 (PMC10189133; doi:10.3389/fimmu.2023.1188018)
Supplement: Supplementary file 1 [file DataSheet_1.docx]

Supplementary Material

Efficient Ex Vivo Expansion of Conserved Element Vaccine-Specific CD8+ T-Cells from SHIV-Infected, ART-Suppressed Nonhuman Primates

Sandra Dross^1,2^, Rasika Venkataraman^3^, Shabnum Patel^4^, Meei-Li Huang^5^,
Catherine M. Bollard^4^, Margherita Rosati^6^, George N. Pavlakis^6^, Barbara K. Felber^7^, Katharine J. Bar^8^, George M. Shaw^8^, Keith R. Jerome^5,9^, James I. Mullins^1,10,11^,
Hans-Peter Kiem^2,3,5,10^, Deborah Heydenburg Fuller^1,2^*, Christopher W. Peterson^3,10^*

*** Correspondence:** Deborah Heydenburg Fuller, [fullerdh@uw.edu](mailto:fullerdh@uw.edu) or Christopher W. Peterson, [cwpeters@fredhutch.org](mailto:cwpeters@fredhutch.org)

# Supplementary Figures 1-4

**Supplementary Figure 1. CE-specific T cell magnitude and polyfunctionality pre-vaccination and pre-CE-XTC manufacturing.** PBMC were isolated at 2 time points described in Figure 1A (Pre-vaccination: prior to the first NHP vaccine dose; Post-vaccination, Pre-expansion: 22 weeks later, immediately prior to initiation of CE-XTC manufacturing) and stimulated overnight with overlapping peptide pools representing sequences for each of 7 or 12 CE of SIV Gag and HIV Env, respectively. Frequencies were measured by flow cytometry following cell surface and intracellular cytokine staining. Shown are the cumulative frequencies of stimulated CD4+ and CD8+ Gag and Env CE-specific T cells expressing IFN-ɣ, IL-2, TNFα and/or CD107a with Granzyme B in pre-vaccination (A) and post-vaccination, pre-expansion (B) PBMC.

**Supplementary Figure 2. Quantification of CFSE-labeled CE-XTC cells in peripheral blood 15 minutes after CE-XTC infusion.** Following infusion of CFSE-labeled CE-XTC in A17044 and A17045, PBMC were isolated and surface stained for Live/dead, CD45, CD3, CD4 and CD8, then analyzed by flow cytometry (background gating shown in A). B-E. CFSE signal was detected in total CD3, CD4 and CD8 T cells in A17044 & A17045 15min post infusion (C, E) at <1% circulating cells and compared to pre-infusion CFSE signal (B, D) for reference.

**Supplementary Figure 3. Lack of CFSE^+^ CE-XTC at study endpoint.** CFSE-labeled CE-XTC were quantified in peripheral blood mononuclear cells (PBMC), mesenteric lymph nodes (MLN) and upper gastrointestinal tract (UGI) of 2 CE-XTC-infused NHP (IDs A17044 and A17045) at necropsy (Nx, 9-12 weeks post-CE-XTC infusion). Overnight-incubated, unstimulated single cell suspensions were surface stained as part of the intracellular staining protocol for Live/dead, CD45, CD3, CD4 and CD8 (background gating for PBMC/MLN shown in A and UGI shown in F) and analyzed by flow cytometry. CFSE+ (infused cells) were not detected in the PBMC (B,D), MLN (C,E) nor UGI (G, H).

**Supplementary Figure 4. CE-specific T cell magnitude and polyfunctionality at study endpoint.** At necropsy (9-12 weeks post-CE-XTC infusion) single cell suspensions from peripheral blood mononuclear cells (PBMC), mesenteric lymph node (MLN) and upper gastrointestinal tract (UGI) were stimulated overnight with overlapping peptide pools representing sequences for each of 7 or 12 CE of SIV Gag and HIV Env, respectively. Frequencies were measured by flow cytometry following cell surface and intracellular cytokine staining. Shown are the cumulative frequencies of stimulated CD4+ and CD8+ Gag and Env CE-specific T cells expressing IFN-ɣ, IL-2, TNFα and/or CD107a with Granzyme B in PBMC (A), MLN (B), and UGI (C). Control animals A17019 and A17020 did not receive CE-XTC prior to SHIV challenge while A17044 and A17045 did receive CE-XTC prior to SHIV challenge.
